# Analyzing isolated degeneration of lumbar facet joints: implications for degenerative instability and lumbar biomechanics using finite element analysis

**DOI:** 10.3389/fbioe.2024.1294658

**Published:** 2024-03-27

**Authors:** Jun Sung Park, Tae Sik Goh, Jung Sub Lee, Chiseung Lee

**Affiliations:** ^1^ Department of Biomedical Engineering, Graduate School, Pusan National University, Busan, Republic of Korea; ^2^ Department of Orthopaedic Surgery, School of Medicine, Pusan National University, Busan, Republic of Korea; ^3^ Biomedical Research Institute, Pusan National University Hospital, Busan, Republic of Korea; ^4^ Department of Biomedical Engineering, School of Medicine, Pusan National University, Busan, Republic of Korea

**Keywords:** lumbar facet joint, isolated degeneration, degenerative instability, lumbar biomechanics, finite element analysis

## Abstract

The facet joint contributes to lumbar spine stability as it supports the weight of body along with the intervertebral discs. However, most studies on the causes of degenerative lumbar diseases focus on the intervertebral discs and often overlook the facet joints. This study aimed to investigate the impact of facet joint degeneration on the degenerative changes and diseases of the lumbar spine. A finite element model of the lumbar spine (L1–S1) was fabricated and validated to study the biomechanical characteristics of the facet joints. To simulate degeneration of the facet joint, the model was divided into four grades based on the number of degenerative segments (L4–L5 or L4–S1) and the contact condition between the facet joint surfaces. Finite element analysis was performed on four spine motions: flexion, extension, lateral bending, and axial torsion, by applying a pure moment to the upper surface of L1. Important parameters that could be used to confirm the effect of facet joint degeneration on the lumbar spine were calculated, including the range of motion (ROM) of the lumbar segments, maximum von Mises stress on the intervertebral discs, and reaction force at the facet joint. Facet joint degeneration affected the biomechanical characteristics of the lumbar spine depending on the movements of the spine. When analyzed by dividing it into degenerative onset and onset-adjacent segments, lumbar ROM and the maximum von Mises stress of the intervertebral discs decreased as the degree of degeneration increased in the degenerative onset segments. The reaction force at the facet joint decreased with flexion and increased with lateral bending and axial torsion. In contrast, lumbar ROM of the onset-adjacent segments remained almost unchanged despite severe degeneration of the facet joint, and the maximum von Mises stress of the intervertebral discs increased with flexion and extension but decreased with lateral bending and axial torsion. Additionally, the facet joint reaction force increased with extension, lateral bending, and axial rotation. This analysis, which combined the ROM of the lumbar segment, maximum von Mises stress on the intervertebral disc, and facet joint reaction force, confirmed the biomechanical changes in the lumbar spine due to the degeneration of isolated facet joints under the load of spinal motion. In the degenerative onset segment, spinal instability decreased, whereas in the onset-adjacent segment, a greater load was applied than in the intact state. When conducting biomechanical studies on the lumbar spine, considering facet joint degeneration is important since it can lead to degenerative spinal diseases, including adjacent segment diseases.

## 1 Introduction

The facet joint is a functional joint that consists of a pair of zygapophyseal joints located at the posterior part of the vertebrae (Gellhorn et al., 2013; Yin et al., 2020). It maintains the stability of spinal motion in the functional spinal unit (FSU) of each vertebral segment and supports approximately 6%–30% of the axial compressive load; therefore, degenerative changes can occur in the facet joint when subjected to excessive loads (Takigawa et al., 2010; Iorio et al., 2015; Yin et al., 2020). Degeneration of the facet joint mainly occurs at the L4–L5 and L5–S1 spinal segments; moreover, pathological changes, such as narrowing of the facet joint space, hypertrophy of the articular process, subchondral cysts, osteophyte formation, and subarticular bone erosions, can be observed on medical images (Weishaupt et al., 1999; Kalichman et al., 2008). The incidence and severity of degeneration tend to increase with age; furthermore, the severity of degeneration is evaluated using the grading system for facet joint degeneration (FJD) developed by Weishaupt et al. (1999), Eubanks et al. (2007).

Degenerative changes in the facet joint can also be accelerated by other spinal disorders, such as degenerative disc disease (DDD) (Fujiwara et al., 1999; Jaumard et al., 2011; Varlotta et al., 2011; Gellhorn et al., 2013). DDD causes a reduction in the height of the intervertebral discs, which increases the compressive load and pressure transmitted to the facet joint, and this eventually damages facet joints (Dunlop et al., 1984; Panjabi et al., 1984; Li et al., 2011). Despite the high incidence of degenerative facet joint disease due to various reasons, relatively few patients with FJD are diagnosed without DDD. Previous studies on FJD have also included patients with degenerative changes in the intervertebral discs. Therefore, conducting studies that focus solely on FJD is difficult.

Recently, a significant increase in the use of computational biomechanics techniques to model and simulate the spine (Fujiwara et al., 2000; Du et al., 2016; Wang et al., 2018; Wang et al., 2019; Song et al., 2021; Kim et al., 2022; Sim et al., 2022) have been reported. Studies focusing on the lumbar facet joints have also been conducted with increasing frequency. Du et al. (2016) investigated the changes in the biomechanical characteristics of the facet joint when various levels of follower preload were applied to the lumbar L1–L5 finite element (FE) model. Wang et al. (2019) developed an FE model of the C5–C6 cervical segment and implemented FJD by modeling the stiffness of the capsular ligament and the angle of the zygapophyseal joint. They also evaluated the biomechanical characteristics of the cervical spine by performing simulations using these parameters and comparing and analyzing the range of motion (ROM) and intradiscal pressure (IDP) of the intervertebral disc. However, confirming the biomechanical characteristics of FJD at each stage was difficult since they were not classified into grades. Regarding the grades of degeneration, Wang et al. (2018) developed a lumbar L1–S1 FE model and performed a series of simulations by adding anterior osteophytes, which are degenerative changes, at each segment. They evaluated the effects on the lumbar spine by grading the anterior osteophytes. Fujiwara et al. (2000) evaluated the biomechanical characteristics of the spine by analyzing the spine motion according to the degeneration grade of the facet joint and intervertebral disc on medical images. Many previous studies have evaluated the effects of degenerative changes in the spine. Nonetheless, there is a paucity of research that effectively differentiates between the grades of degeneration specifically within the lumbar facet joint, excluding considerations of intervertebral disc degeneration. Moreover, there is a dearth of studies assessing the biomechanical repercussions of lumbar facet joint degeneration on the lumbar spine.

Therefore, this study focused only on lumbar FJD. Using finite element analysis, we aimed to confirm the effects of various levels of lumbar FJD on lumbar activity. In order to simulate diverse degrees of degeneration, we utilized the grading system for FJD as a reference and assumed that the gap and friction coefficient of the articular surface were key variables (Weishaupt et al., 1999). We classified the lesions into five levels based on this grading system: intact state (grade 0; G0), mild (Grade 1; G1), mild-moderate (Grade 2; G2), moderate (Grade 3; G3), and severe generation (Grade 4; G4). In addition, we considered two cases with different numbers of degenerated segments, considering the location of the segments where degeneration mainly occurs in the facet joint: a single segment at the L4–L5 and double segments at the L4–S1 segments. Both of these correspond to the onset segment. And the onset adjacent segments are L3-L4 (upper segment), L5-S1 (lower segment) for single segment facet joint degeneration and L3-L4 (upper segment) for double segments facet joint degeneration. In this study, we aimed to strategically evaluate the impact of the degeneration grade and the number of onset segments on onset and onset-adjacent segments.

## 2 Materials and methods

**FIGURE 1:**
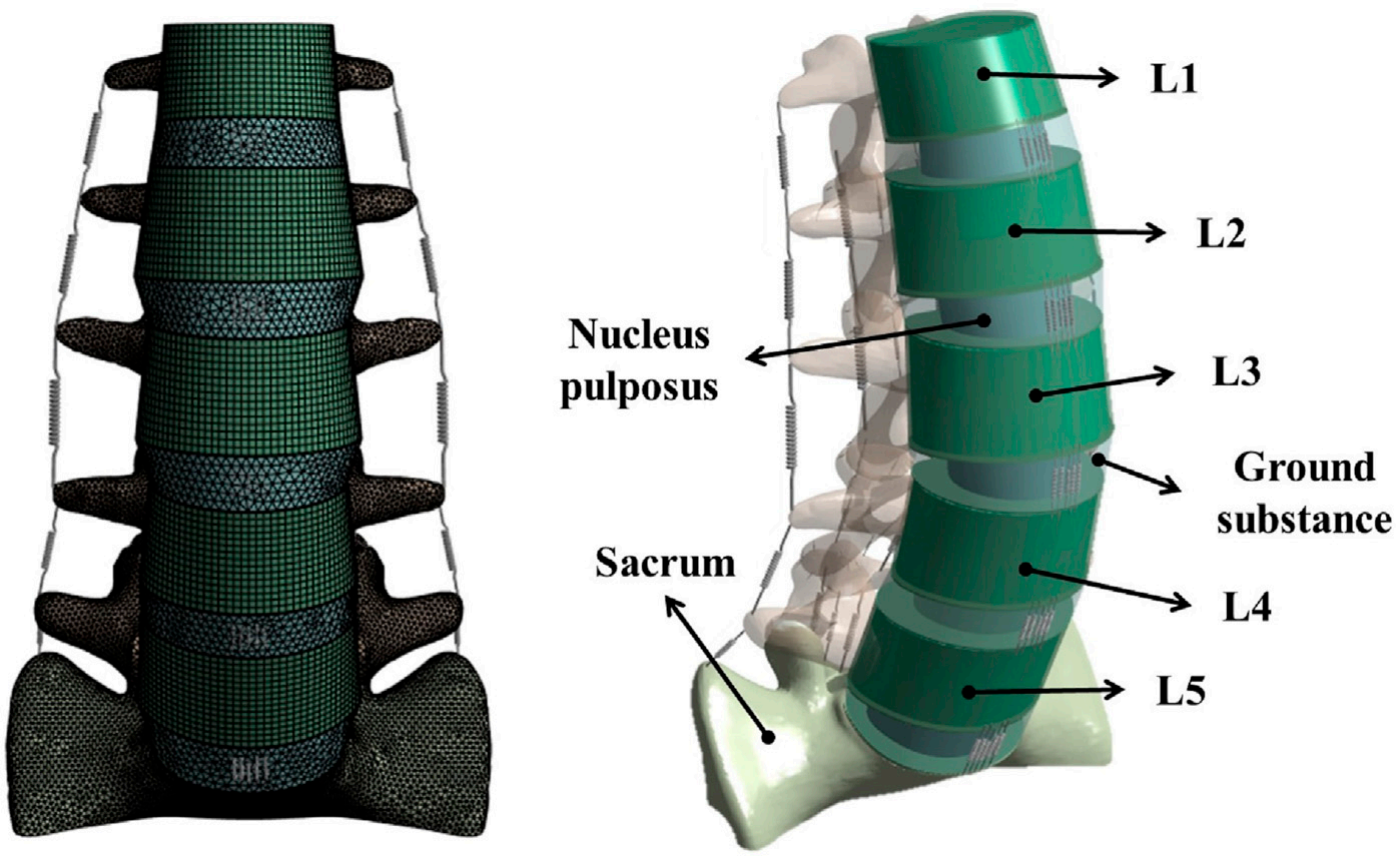
Finite element model of the lumbar spine including the L1–S1 spinal segment with vertebral bodies, intervertebral discs, and ligaments.

The normalized shape of the lumbosacral spine (L1–S1) was extracted from computed tomography (CT) images of the spine of a 28-year old male (80 kg, 178 cm) without metabolic bone disease using Mimics 21.0 (Materialise, Leuven, Belgium). The shape of the vertebral body (L1–S1) was extracted using Geomagic Design X software (3D Systems, Rock Hill, SC, United States). Afterwards, noise and defects that occurred during shape extraction were removed from the medical images. These images were then exported as an STP file for additional detailed modeling using the Inventor 2018 (Autodesk, Mill Valley, CA, United States). The tasks carried out with Inventor included simplification of the vertebral body and additional modeling of intervertebral discs (annulus fibrosus and nucleus pulposus) and endplates to create a 3D geometric model, as shown in Figure 1.

The generated 3D model was subjected to FEA preprocessing on an ANSYS Workbench 2022 R2 (ANSYS Inc. Canonsburg. PA. United States), a commercial finite element analysis program. The material properties of each component of the 3D model are listed in Table 1 (Goel et al., 1995; Lu et al., 1996; Rohlmann et al., 2006; Rohlmann et al., 2009; Liu et al., 2011; Dreischarf et al., 2014; Du et al., 2014; Nikkhoo et al., 2020; Mengoni, 2021). Among the components of the FE model, vertebral bodies (cortical bone, cancellous bone, and posterior elements) and endplates were adopted as isotropic linear material properties. The cortical bone, cancellous bone, and endplate were hexahedral elements, and the posterior elements were tetrahedral elements. The annulus fibrosus and incompressible nucleus pulposus of the intervertebral disc were adopted as hyperelastic material models using Mooney Rivlin with tetrahedral elements. Additionally, seven representative ligaments were defined as nonlinear materials using tension-only spring elements: the anterior longitudinal ligament, posterior longitudinal ligament, interspinal ligament, supraspinal ligament, intertransverse ligament, ligamentum flavum, and capsular ligament (Table 2). Based on previous studies, the modeling of the intact facet joint assumes certain parameters, including a frictionless interface, an initial gap of 0.5 mm, an exponential formulation, and a pressure of 120 MPa at a zero gap (Liu et al., 2020; Mengoni, 2021). A finite element model (Model0; M0) of the intact state of lumbar L1–S1 was constructed, as shown in Figure 1.

**TABLE 1:** Material properties used in the developed FE model.

Components	Young’s modulus (MPa)	Poisson’s ratio	References
Cortical bone	10,000	0.3	Rohlmann et al. (2009)
Cancellous bone	100	0.2	Goel et al. (1995)
Posterior bone	3500	0.25	Goel et al. (1995)
Sacrum	5000	0.2	Du et al. (2014)
Endplate	23.8	0.4	Lu et al. (1996)
Ground substance	Mooney-Rivlin C10 = 0.42, C01 = 0.105		Liu et al. (2011)
Nucleus pulposus	Mooney-Rivlin C10 = 0.12, C01 = 0.03		Du et al. (2014)
Ligaments	Nonlinear stress-strain curve		Rohlmann et al. (2006)

**TABLE 2:** Properties of the ligaments (Rohlmann et al., 2006).

Ligament	Stiffness (K1) (N/mm)	Strains (ε1) (−)	Stiffness (K2) (N/mm)	Strains (ε2) (−)	Stiffness (K3) (N/mm)
ALL	347	0.122	787	0.203	1864
PLL	29.5	0.111	61.7	0.230	236
ISL	1.4	0.139	1.5	0.200	14.7
SSL	2.5	0.200	5.3	0.250	34
ITL	0.3	0.182	1.8	0.233	10.7
LF	7.7	0.059	9.6	0.490	58.2
CL	36	0.250	159	0.300	384

To validate the FE model constructed in this study, we compared and analyzed the ROM of L1–S1 under the following conditions: flexion of 8 Nm, extension of 6 Nm, lateral bending of ±6 Nm, and axial torsion of ±4 Nm, which were the same as the spine motion in previous studies. We also compared and analyzed the IDP under a compressive follower load and the height of the intervertebral disc between the L4–L5 vertebrae at a compressive follower load of 1,200 N (Renner et al., 2007; Dreischarf et al., 2014).

**FIGURE 2:**
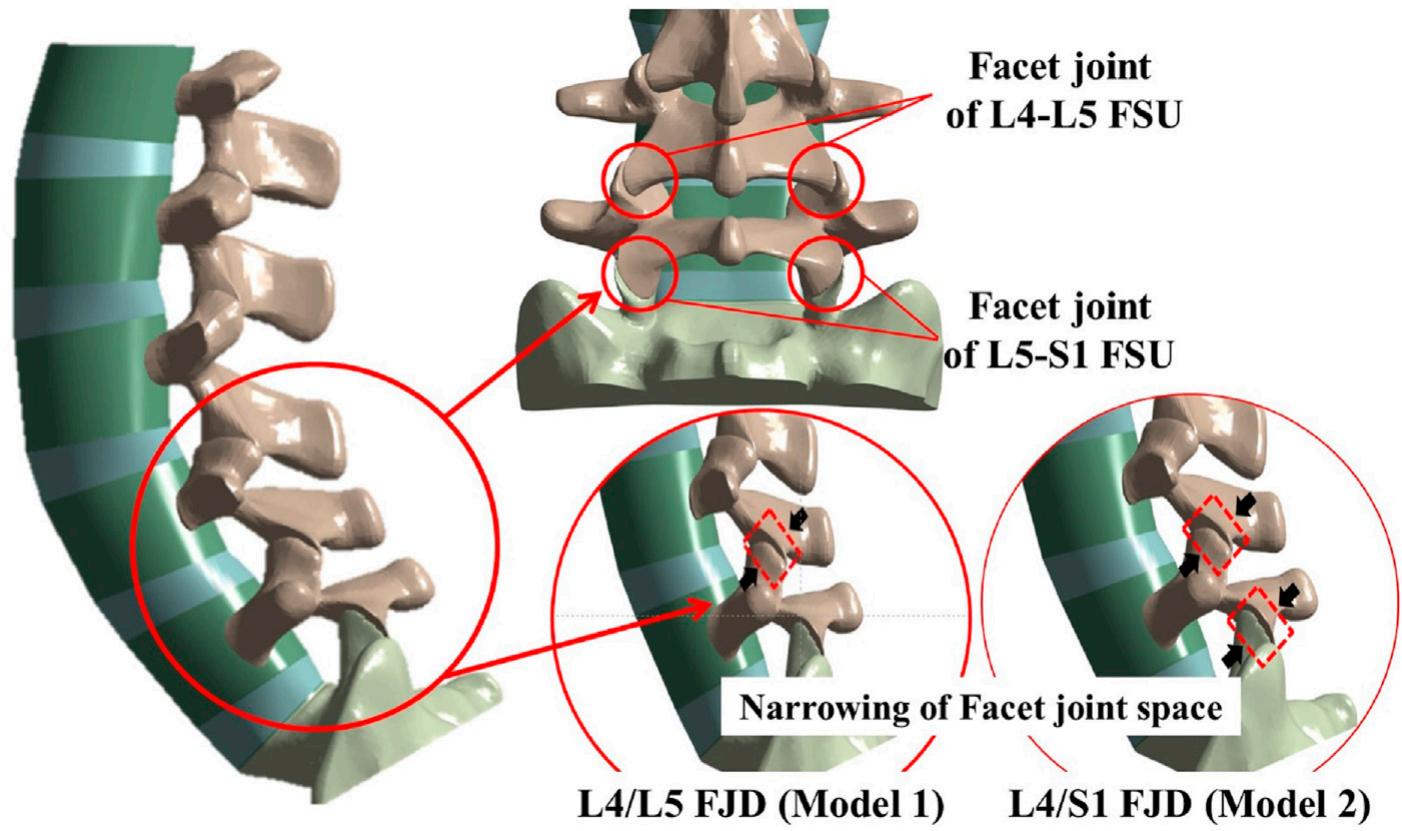
Localization of facet joint degeneration and modified facet joint space narrowing method at L4–L5 and L5–S1 functional spinal units (FSUs).

The location and number of degenerative segments of facet joints were the main variables in this study and were represented by L4/L5 FJD (Model1; M1) and L4/S1 FJD (Model2; M2), respectively. The degeneration grade of the facet joint was classified into five stages, including the intact state, based on the severity of degeneration. The gap and friction coefficient of the contacting surface of the facet joint were assumed to be graded based on the stage of degeneration, where Grade 0 corresponds to 100% and Grade 4 corresponds to 10%, with intermediate grades of 75, 50, and 25% corresponding to Grade 1, 2, and 3, respectively (Table 3). Finally, the gap in the contacting surface of the facet joint in L4–L5 FSU and L5–S1 FSU according to the degenerative location and degree of the facet joint is shown in Figure 2.

**TABLE 3:** Primary variables associated with lumbar facet joint degeneration and the values of each primary variable based on the degree of degeneration.

Degree of FJD	Lumbar facet joint contact parameters	
	Facet joint gap size	Friction coefficient
Grade0 (G0)	0.5 mm (100%)	0 (100%)
Grade1 (G1)	0.375 mm (75%)	0.25 (75%)
Grade2 (G2)	0.25 mm (50%)	0.5 (50%)
Grade3 (G3)	0.125 mm (25%)	0.75 (25%)
Grade4 (G4)	0.05 mm (10%)	0.9 (10%)

## 3 Results

### 3.1 Validation of the finite element (FE) model

**FIGURE 3:**
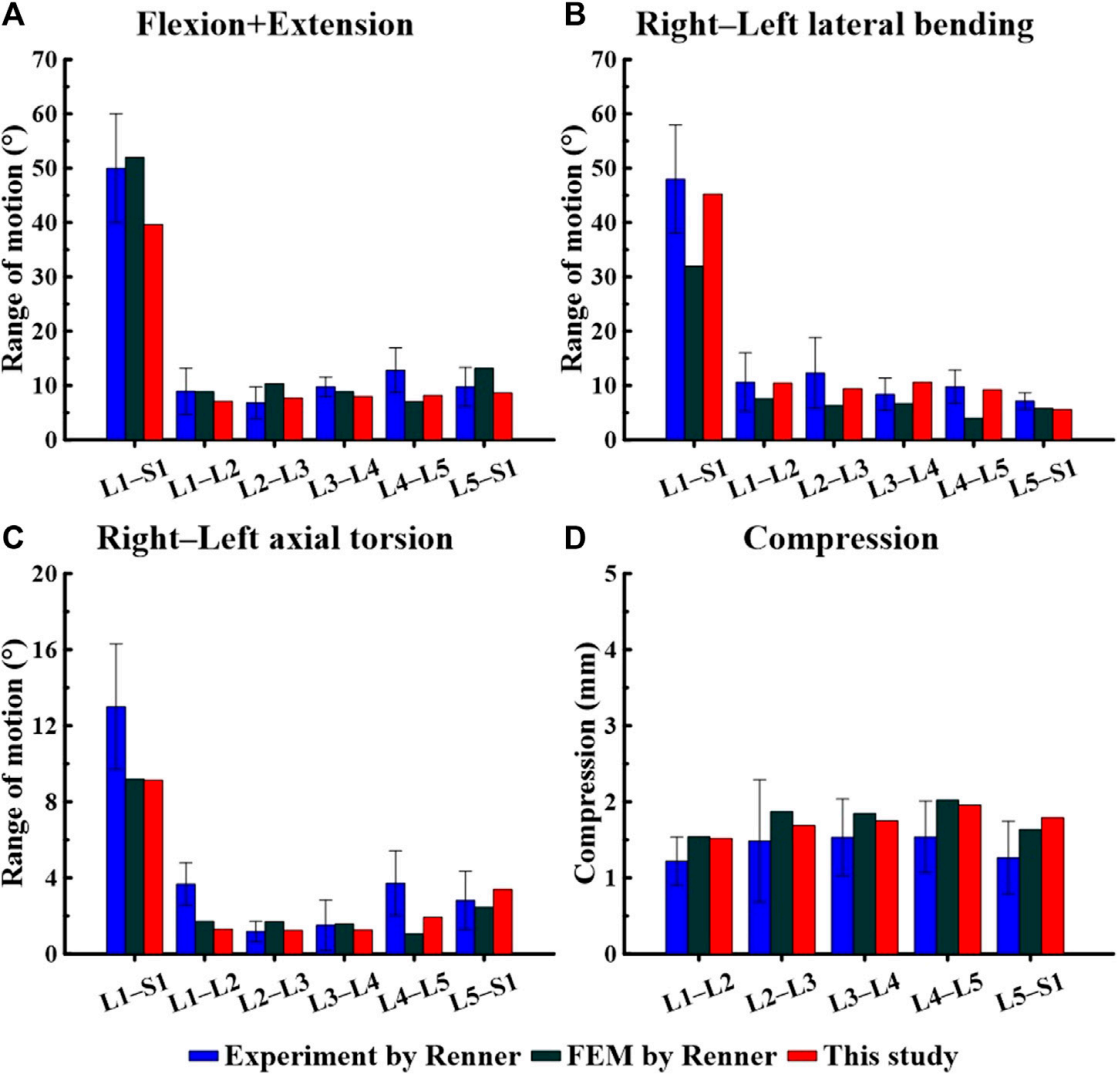
Comparison of the range of motion (ROM) based on spinal motion between the model used in this study and that of a study by Renner et al. (2007). **(A)** Flexion + Extension, **(B)** Right–Left lateral bending, **(C)** Right–Left axial torsion, **(D)** Compression.

**FIGURE 4:**
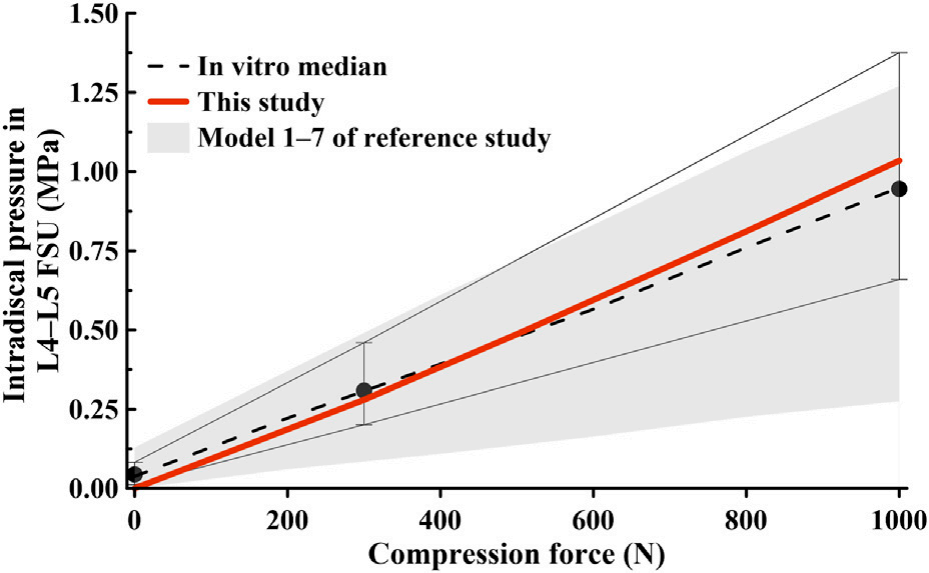
Comparison of intradiscal pressure (IDP) findings at the L4–L5 functional spinal unit (FSU) in this study and those in previous studies (Dreischarf et al., 2014).

The FE model was validated before conducting the study. We compared the measured ROM during spine movements (flexion, extension, lateral bending, and axial torsion) in Figures 3, 4, and checked whether they were within the range of results from previous studies. We also compared the IDP at the L4–L5 FSU and the height of the intervertebral disc at the L4–L5 FSU under follower load with the results of previous studies. The ROM and intervertebral disk height measured in the FE model developed in this study were compared with the results of the study by Renner et al. (2007) on *ex vivo* spinal structures and spinal FE model behavior simulations. We confirmed that the ROM of the entire segment (L1–S1) and each segment measured in this study were within the range of results reported by Renner et al. Additionally, when comparing IDP at the L4–L5 FSU, our model was also within the range of IDP from seven previous studies on spine motion simulations and *ex vivo* spinal structure experiments (Dreischarf et al., 2014). Based on this, we concluded that the lumbar L1–S1 FE model developed in this study can be utilized.

### 3.2 Range of motion (ROM)

**FIGURE 5:**
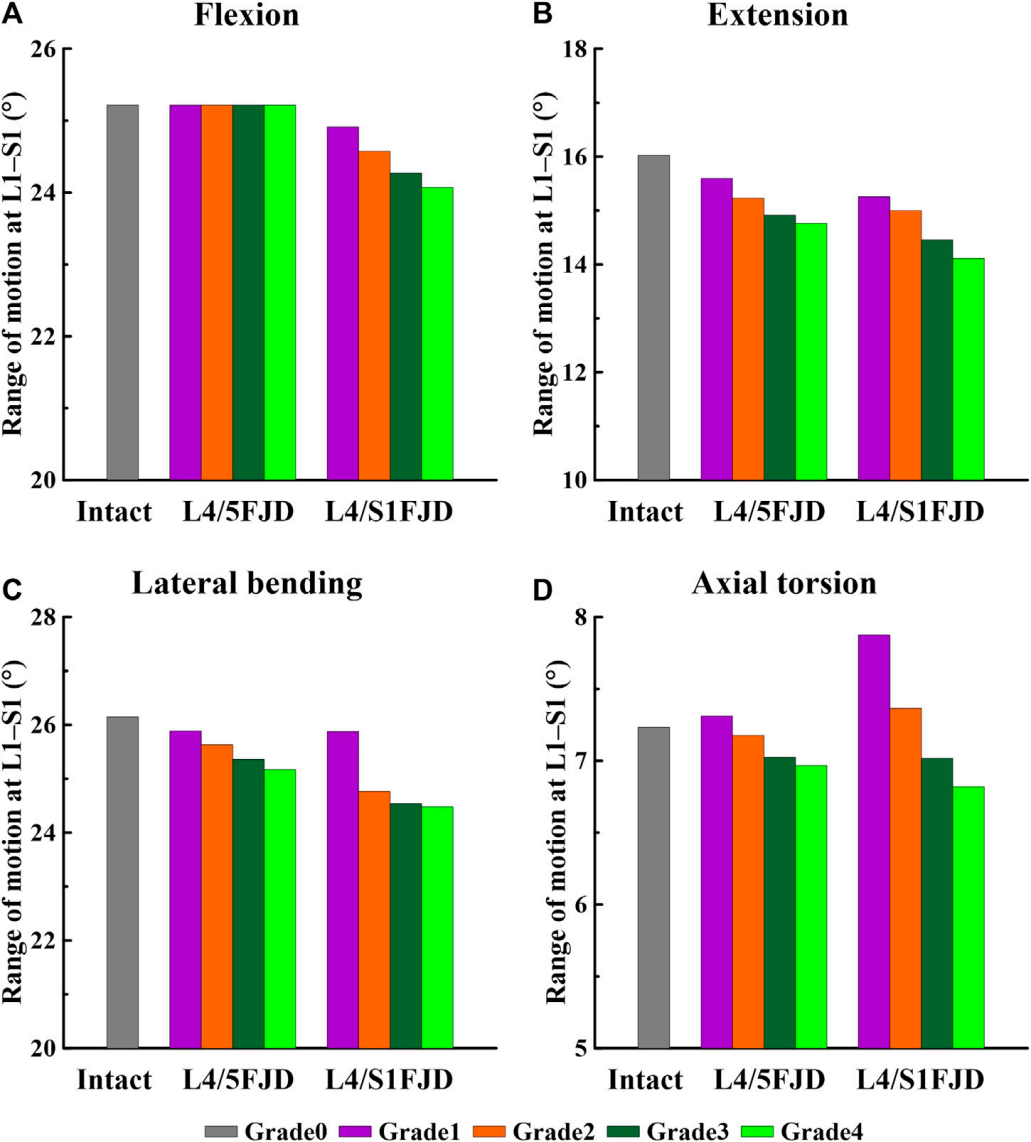
Comparison of the total range of motion (ROM) based on the degree of lumbar facet joint degeneration. **(A)** Flexion, **(B)** Extension, **(C)** Lateral bending, **(D)** Axial torsion.

**FIGURE 6:**
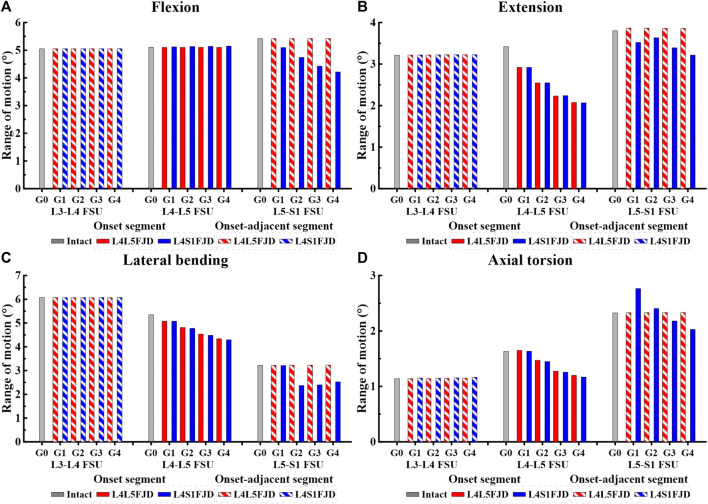
Comparison of the range of motion (ROM) in onset and onset-adjacent segments (L3–L4, L4–L5, and L5–S1) according to the grade of lumbar facet joint degeneration. **(A)** Flexion, **(B)** Extension, **(C)** Lateral bending, **(D)** Axial torsion.

Figures 5, 6 show the results of the lumbar segmental ROM simulations performed under the loading conditions described in Table 4. Specifically, Figure 5 shows the total ROM of lumbar L1–S1, and Figure 6 shows the ROM of the degenerated and adjacent segments of the facet joint.

**TABLE 4:** Loading conditions applied to L1 vertebra in this study.

Spinal motion	Flexion	Extension	Lateral bending	Axial torsion
Moment (Nm)	7.5	7.5	7.5	7.5

As shown in Figure 5, as the degeneration of the facet joint worsened in all spine motions, the ROM of the L1–S1 vertebrae gradually decreased. Furthermore, when degeneration occurred in two segments (L4/S1 FJD; M2) rather than one segment (L4/L5 FJD; M1), the ROM of the L1–S1 vertebrae decreased. During flexion movements, Model 1 did not differ significantly from Model 0 in terms of ROM, even as the degeneration grade increased. Model 2 decreased by approximately 1.2%–4.5% compared to Model 0 (Figure 5A). During extension movements, both Models 1 and 2 decreased the ROM compared to Model 0, while Model 2 showed a maximum decrease of 11.9% (Grade 4) (Figure 5B). During lateral bending movements, both Models 1 and 2 decreased the ROM compared to Model 0, whereas Model 2 decreased by 6.4% (Grade 4) compared to Model 0 (Figure 5C). Moreover, during axial torsion movements, Model 1 increased by 1.1%–8.9% in Grade 1, and Model 3 increased by Grade 1–2 compared to Model 0. However, as the degree of FJD increased, the ROM of Model 0 decreased compared with that of Model 1 in Grade 1 and Model 3 in Grade 1–2 (Figure 5D).

The ROM of each segment (L4–L5 FSU, L5–S1 FSU) decreased gradually as the degeneration of the facet joint worsened in all spinal motions, as shown in Figure 6. However, the ROM of each segment (L3–L4 FSU, L5–S1 FSU) in adjacent segments was not significantly different from that of Model 0 in an intact state. In the initial analysis of the ROM for each segment (L4–L5 FSU, L5–S1 FSU) in the onset segment, the ROMs of Models 1 and 2 remained nearly identical during the flexion movement within the L4–L5 FSU. Furthermore, the ROM did not change significantly even as the grade of degeneration increased (Figure 6A). The ROM difference between the two models was almost negligible during other movements, such as extension, lateral bending, and axial torsion; however, the ROM gradually decreased depending on the degeneration grade, unlike during flexion movement. Compared to the ROM of Model 0, the ROM reduction rates of the two models were 14.62%–39.28% (M1G1–M1G4) and 14.59%–39.51% (M2G1–M2G4) during extension, and 5%–18.81% (M1G1–M1G4) and 5%–19.71% (M2G1–M2G4) during lateral bending (Figures 6B,C). Furthermore, during axial torsion, the ROM slightly increased in Grade 1 but showed a reduction rate of 26.43% (M1G4) and 28.33% (M2G4) in Grade 4 (Figure 6D). In the L5–S1 FSU as shown in Figure 6, Model 2 showed a gradual decrease in ROM depending on the grade of degeneration in all spine motions (flexion, extension, lateral bending, and axial torsion), and the maximum reduction rates in each movement observed in Grade 4 were 22.24 (flexion), 15.43 (extension), 21.53 (lateral bending), and 12.87% (axial torsion). Finally, regarding the ROM of each segment (L3–L4 FSU, L5–S1 FSU) in adjacent segments, the ROMs of Models 1 and 2 showed the same values, regardless of the degeneration grade, and were almost identical to the ROM of Model 0.

### 3.3 Maximum von mises stress of the intervertebral disc

**FIGURE 7:**
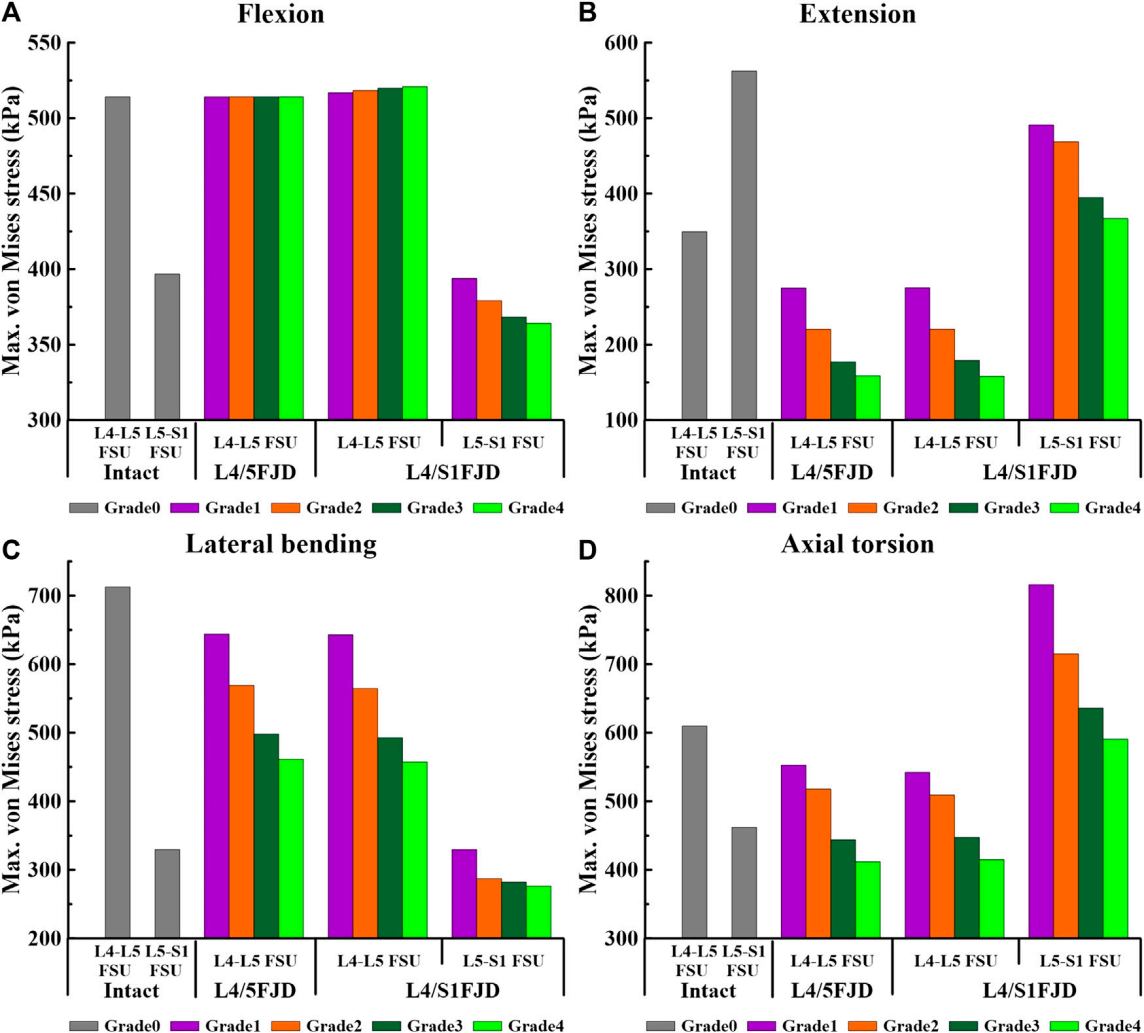
Comparison of the maximum von Mises stress in intervertebral discs at the onset segment based on the degree of lumbar facet joint degeneration and spinal motion (flexion, extension, lateral bending, and axial torsion). **(A)** Flexion, **(B)** Extension, **(C)** Lateral bending, **(D)** Axial torsion.

**FIGURE 8:**
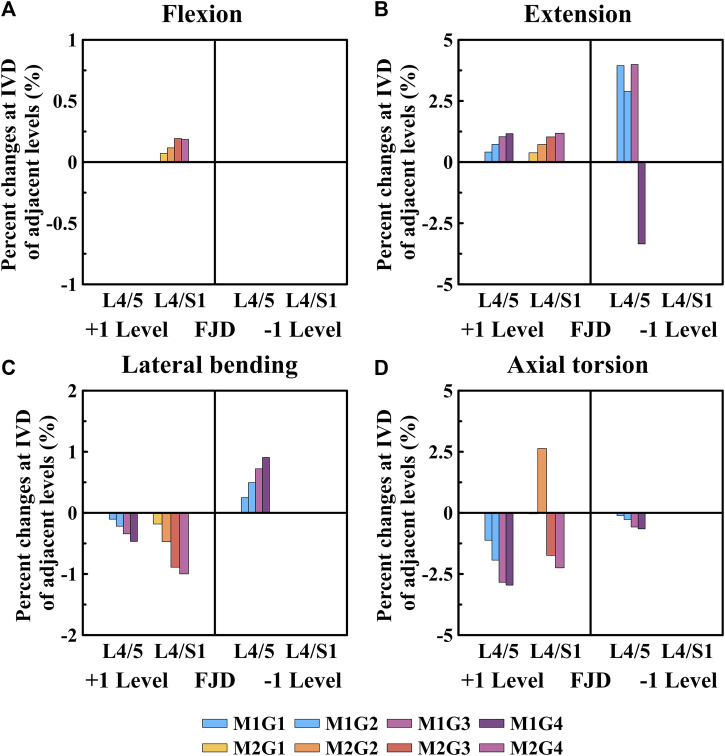
Comparative analysis of the percentage changes in maximum von Mises stress at the intervertebral discs of adjacent levels compared to that of the intact state, based on the degree of lumbar facet joint degeneration and spine motion (flexion, extension, lateral bending, and axial torsion). **(A)** Flexion, **(B)** Extension, **(C)** Lateral bending, **(D)** Axial torsion. Percentage change = (data of Model1 or Model2–data of Model0)/data of Model0×100%.

Figures 7, 8 show the maximum von Mises stress results of the simulations performed under the load conditions listed in Table 4 for the intervertebral discs. Specifically, Figure 7 shows the maximum von Mises stress of the intervertebral disc at the onset segment, whereas Figure 8 shows the maximum von Mises stress of the intervertebral disc in the adjacent upper and lower segments at the onset; both are presented as a percentage change from the intact state (M0).

As shown in Figure 7, the maximum von Mises stress on the intervertebral disc decreased as the degeneration of the facet joint increased in all spinal motions. Moreover, in Model 2, which comprises two segments, there was a notably higher maximum von Mises stress observed on the L5–S1 FSU during axial rotation when compared to that of Model 0. However, the trend of gradually decreasing maximum von Mises stress on the intervertebral disc according to the degeneration grade was consistent. Firstly, regarding the maximum von Mises stress of the intervertebral disc at onset, it was observed that Model 1 and Model 2 increased slightly or remained the same as Model 0 during flexion movement in L4–L5 FSU, and that Model 2 increased by 4.04 kPa–520.9 kPa (M2G4) in Grade 4. Furthermore, regarding the L5–S1 FSU, Model 2 gradually decreased as degeneration increased, showing a decrease rate of 0.74%–8.23% (M2G1–M2G4) compared to Model 0 (Figure 7A). Furthermore, considering extension, lateral bending, and axial torsion movements (Figures 7B–D), when compared to Model 0, the decreased rate in Model 2 in the L4–L5 FSU was 21.29%–54.60% (M1G1–M1G4) during extension, 21.18%–54.72% (M2G1–M2G4) during lateral bending, and 9.34%–32.49% (M1G1–M1G4) and 11.10%–31.95% (M2G1–M2G4) during axial torsion. In the L5–S1 FSU, the decreased rate in Model 2 was 12.69%–34.75% (M2G1–M2G4) during extension and 0.00%–16.15% (M2G1–M2G4) during lateral bending. However, it increased compared to Model 0 during axial torsion, showing an increase rate of 27%–76% (M2G4–M2G1).

As shown in Figure 8, the percentage change in the maximum von Mises stress of the intervertebral disc in a single segment adjacent to the affected area gradually increased during flexion and extension movements as the degeneration of the facet joint worsened compared to the intact state (M0) (Figures 8A, B). Additionally, the percentage change in the maximum von Mises stress of the intervertebral disc gradually decreased during lateral bending and axial torsion movements (Figures 8C, D). First, when observing the adjacent upper segment, there was no change in Model 1 as the grade of degeneration of the facet joint increased during flexion movements; furthermore, in Model 2, it only slightly increased, with a maximum increase rate of 0.18% (M2G4), which was not statistically significant (Figure 8A). During extension, the maximum increase rates of Model 1 and Model 2 were slightly increased to 1.16% (M1G4) and 1.18% (M2G4), respectively (Figure 8B). In the lateral bending and axial torsion movements, the maximum decrease rates of Model 1 and Model 2 were 0.46% (M1G4) and 1.00% (M2G4) for lateral bending and 2.95% (M1G4) and 2.25% (M2G4) for axial torsion, respectively (Figures 8C,D). Furthermore, when observing the adjacent lower segment, which was the L5–S1 FSU, only Model 1 was observed. During the extension movements, it increased to approximately 4.00% up to Grade 3, depending on the degeneration grade of the facet joint, and then decreased to approximately 3.34% at Grade 4 (Figure 8B). The maximum increase and decrease rates during the lateral bending and axial torsion movements were 0.90% (M1G4) and 0.65% (M1G4), respectively, depending on the degeneration grade of the facet joint (Figures 8C,D).

### 3.4 Reaction force on the facet joint

**FIGURE 9:**
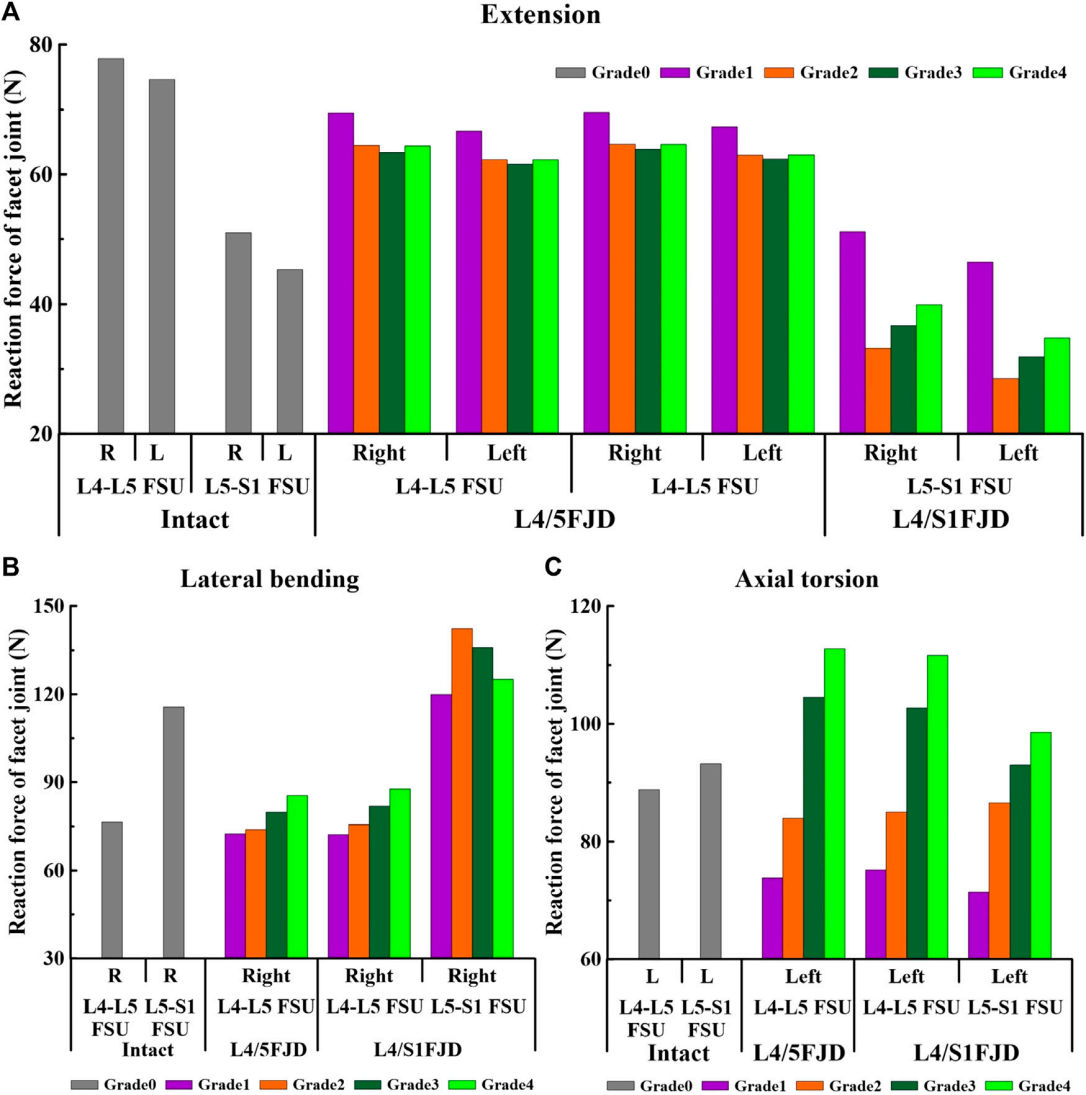
Comparison of the reaction force in the facet joint at the onset segment based on the degree of lumbar facet joint degeneration and spine motion. **(A)** Extension, **(B)** Lateral bending, **(C)** Axial torsion.

**FIGURE 10:**
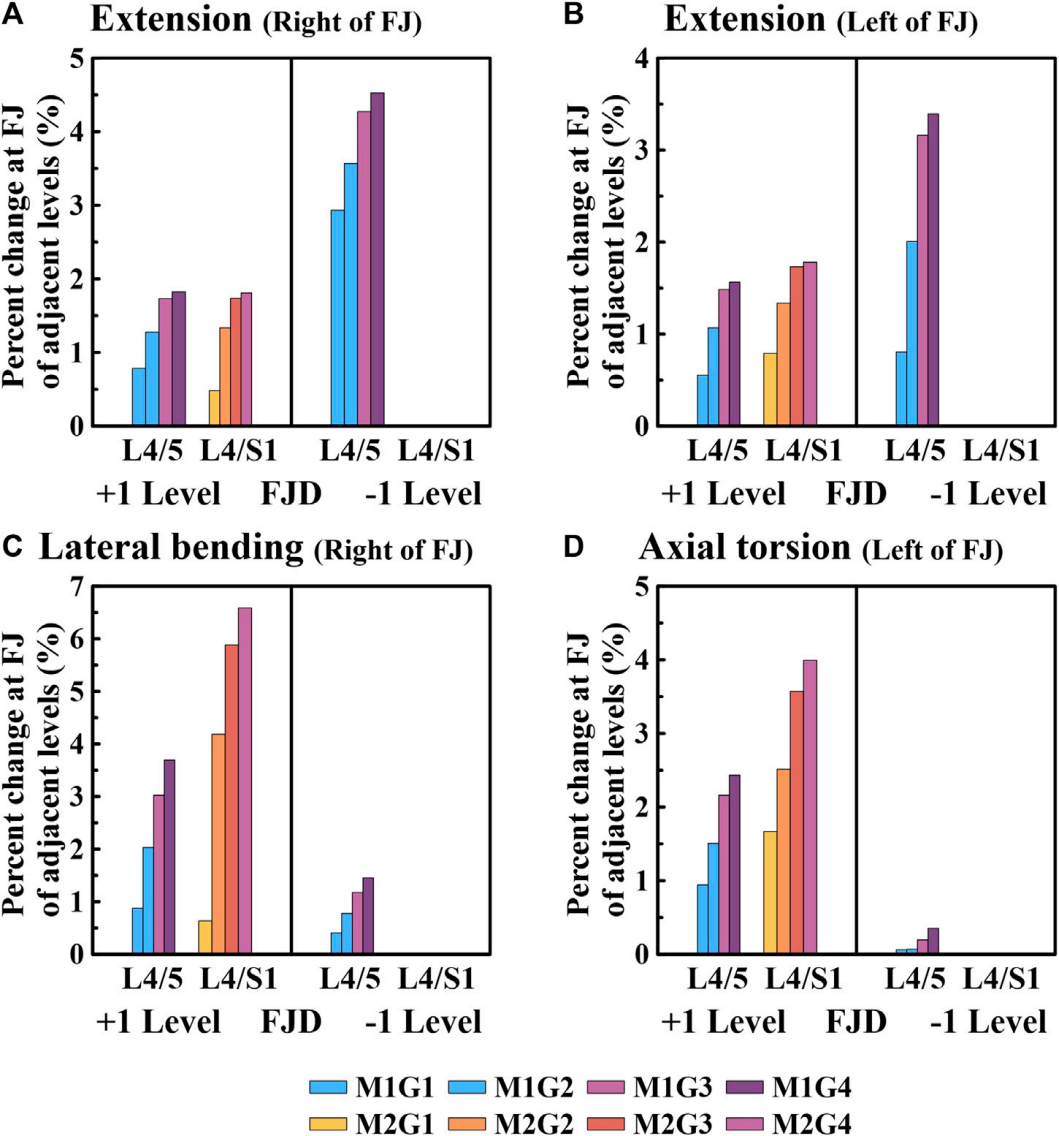
Comparative analysis of the percentage changes in the reaction force at the facet joints of adjacent levels compared to the intact state based on the degree of lumbar facet joint degeneration and spine motion. **(A)** Right of facet joint during extension, **(B)** Left of facet joint during extension, **(C)** Right of facet joint during lateral bending, **(D)** Left of facet joint during axial torsion. Percentage change = (data of Model1 or Model2–data of Model0)/data of Model0×100%.

Figures 9, 10 present the reaction force results of the simulations performed under the load conditions of the facet joints listed in Table 4. Specifically, Figure 9 shows the reaction force of the facet joint at the onset segment, whereas Figure 10 shows the reaction force of the facet joint in the adjacent upper and lower segments at the onset. Both are presented as a percentage change from the intact state (M0). However, flexion movements that did not cause a facet joint reaction force because the articular surfaces of the zygapophyseal joints that did not come into contact during spinal motion were excluded (Cai et al.). In addition, only the right direction was implemented for lateral bending and axial torsion movements, and the right zygapophyseal joint was excluded when the gap between the zygapophyseal joints widened during right lateral bending and right axial torsion movements.

First, regarding the facet joint reaction force in the onset segment, the forces of both the left and right facet joints during spinal motion showed a similar trend depending on the degeneration grade of the facet joint. During the simulated extension movements (Figure 9A), the facet joint reaction force in both Models 1 and 2 decreased and then increased depending on the degeneration of the onset segments. In Model 1, at L4–L5 FSU, the facet joint reaction force decreased from 69.496 N to 66.68 N (M1G1) to 63.398 N and 61.585 N (M1G3), and increased to 64.389 N and 62.263 N (M1G4). In Model 2, at the L4–L5 FSU, the facet joint reaction force decreased from 69.566 N to 67.343 N (M2G1) to 63.887 N and 62.378 N (M2G3), and increased to 64.623 N and 63.028 N (M2G4), while at the L5–S1 FSU, it decreased from 51.152 N to 46.467 N (M2G1) to 33.191 N and 28.509 N (M2G2) and increased to 39.927 N and 34.801 N (M2G4). During the simulated lateral bending movements (Figure 9B), the facet joint reaction force showed an increasing trend depending on the degeneration of the onset segments in the L4–L5 FSU and a decreasing trend in the L5–S1 FSU. In Model 1, at the L4–L5 FSU, the facet joint reaction force gradually increased from 72.516 N (M1G1) to 85.46 N (M1G4). In Model 2, at L4–L5 FSU, the facet joint reaction force gradually increased from 72.256 N (M2G1) to 87.673 N (M2G4), and at L5–S1 FSU, it increased from 119.93 N (M2G1) to 142.26 N (M2G2) and then decreased to 125.1 N (M2G4). Owing to the degeneration of the facet joint during the axial torsion movement (Figure 9C), the facet joint reaction force gradually increased in the L4–L5 and L5–S1 FSUs. Model 1 gradually increases from 73.83 N (M1G1) to 112.81 N (M1G4) in the L4–L5 FSU. Model 2 showed an increase from 75.189 N (M2G1) to 111.67 N (M2G4) in the L4–L5 FSU and an increase from 71.47 N (M2G1) to 98.564 N (M2G4) in the L5–S1 FSU.

We confirmed that the percentage change in the facet joint reaction force gradually increased during all spinal motions compared with that of the intact facet joint (M0) in the adjacent upper and lower segments where the onset occurred (Figure 10). Moreover, in the case of onset in both segments (M2), the increase was greater than that in the case of onset in one segment (M1). During extension movements (Figures 10A, B), the percentage change in the reaction force compared to the intact (M0) left and right facet joints in the adjacent upper segment gradually increased as the degeneration grade of the facet joint increased, reaching a maximum of 1.82% and 1.56% (M1G4), 0.48% and 0.53% (M2G4), and 1.81% and 1.78% (M3G4). In addition, during adjacent lower segment movements the percentage change in the reaction force of the left and right facet joints gradually increased, similar to that of the adjacent upper segment, ranging from 2.9% to 4.52% and 0.8%–3.39% (M1G1–M1G4). During lateral bending movements (Figure 10C), the percentage change in reaction force compared with that of the intact facet joint (M0) in the adjacent upper segment gradually increased as the degeneration grade of the facet joint increased, reaching a maximum of 3.7% (M1G4) and 6.5% (M2G4). In addition, even in the facet joint of the adjacent lower segment, a maximum of 1.45% (M1G4) was observed. During axial torsion movements (Figure 10D), the percentage change in the reaction force compared with that of the intact (M0) facet joint in the adjacent upper segment gradually increased as the degeneration grade of the facet joint increased, reaching a maximum of 2.43% (M1G4) and 3.99% (M2G4). The change in percentage compared to the intact right facet joint of the adjacent lower segment was minimal, with a maximum of 0.35% (M1G4).

## 4 Discussion

In the present study, the biomechanical effects of FJD on the lumbar spine were investigated through finite element analysis. Finite element models with varying degrees of FJD and numbers of onset segments were fabricated to simulate four common physiological loads experienced during daily activities. The degree of FJD affected the ROM of the lumbar spine, von Mises stress of the intervertebral discs, and facet joint reaction force, demonstrating how FJD affects onset and onset-adjacent segments. As the grade of FJD increased during spinal motion, the ROM gradually decreased, particularly in the onset segments. Model 2, where FJD occurred in two segments, showed a much greater decrease in ROM than did Model 1. Similarly, in each segment with FJD, the decrease in ROM was similar to or slightly greater in Model 2 than in Model 1, demonstrating the influence of the number of onset segments on the decrease in ROM. However, the number of onset segments did not have a significant effect on the maximum von Mises stress of the intervertebral discs or facet joint reaction force.

In a recent study (Yin et al., 2020), medical images of patients with degenerative lumbar facet joint disease who experienced spine motion after maximum bending of the body during flexion-extension, lateral bending, and axial torsion were captured using a dual fluoroscopic imaging system. Based on this, a 3D model of the L3–S1 spinal segment was developed, and the ROM of each was measured. As a result, the ROM was found to be highest in the moderate stage of spinal motion but decreased in the severe stage. These results differ from the tendency of ROM to gradually decrease with FJD, as reported in previous studies. This is attributed to difficulty in completely controlling for variables, such as the possibility that patients with degenerative facet joint disease may also experience degeneration of the IVDs in previous studies. However, both studies showed that ROM decreases during spinal motion in patients with severe FJD, indicating that degenerative facet joint disease limits spinal motion.

In a study conducted by Fujiwara et al. (2000), intervertebral discs and facet joints of patients were graded according to the degeneration, and their ROM was compared based on the motion of their spines. Specifically, degenerative changes in the facet joints were categorized into cartilage degeneration, osteophyte formation, and osteoarthritis, each of which was further divided into four grades to compare the ROM. Osteoarthritis, one of the three symptoms of facet joint degeneration, exhibits a gradual decrease in ROM as degeneration worsens, which is similar to the findings of the current study. Since the main parameters of this study were assumed to be the gap and friction coefficient of the posterior joint, we believe that the results are similar to those of osteoarthritis, where the articular surface of the facet joint becomes rougher as the joint space narrows (Jarraya et al., 2018).

In addition, it has been reported that degenerative changes in the facet joint due to osteoarthritis can cause friction between the bones of the facet joint (Jarraya et al., 2018; Anastasia et al., 2022). This means that as the grade of degeneration of the facet joint increases, a higher coefficient of friction can occur between the bones. However, it has been difficult to formalize this. In this study, we assumed four grades of degeneration and a constant coefficient of friction for each grade. This way, we aimed to emulate the interosseous friction that occurs in clinical degeneration of the facet joint. 

Spinal degeneration occurs naturally with aging and is further accelerated by mechanical weakening of tissues, such as ligaments, due to repetitive injuries or trauma (Jaumard et al., 2011; Wang et al., 2019). This degenerative process is characterized by temporary functional impairment, instability, and restabilization (Kirkaldy-Willis and Farfan, 1982). Additionally, it has been reported that the role of facet joints changes as lumbar degeneration progresses (Kim et al., 2019), leading to degenerative changes in these joints due to excessive load, which increases lumbar instability. The role of facet joints is considered to be as critical as that of intervertebral discs in lumbar degeneration. Biomechanically, facet joints also play a role in limiting axial torsion and are involved in rotational kinematic mechanisms. Consequently, the stages of FJD are thought to be observable in the axial torsion behavior (Adams and Hutton, 1983; Kalichman and Hunter, 2007).

In this study, we observed instability and restabilization of the lumbar spine during axial torsion. Specifically, in the case of the L5–S1 FSU, which is the onset segment, Model 2 exhibited a higher ROM than Model 0 in Grade 1, indicating lumbar instability. As Grade 4 was reached, the ROM decreased compared to that of Model 0, indicating lumbar restabilization. We confirmed that as the ROM of the onset segment decreased during the restabilization stage, the load on the intervertebral disc caused by the onset segment was significantly reduced. Notably, the average decrease rate in the L4–L5 FSU of both models showed a reduction of 54% (flexion), 35% (lateral bending), and 32% (axial torsion) in Grade 4 compared to those of the intact state. However, in the facet joints of the onset segment, the load gradually increased as the degeneration grade increased. For Grade 4, the average increase rates of the two models were 12.5% (lateral bending) and 26% (axial torsion) compared with the intact state. Through this research, it is possible to quantitatively check the information that is difficult to check in medical images, such as the stress on the intervertebral disc and the reaction force of the facet joint, in addition to the information that can be checked in medical images during the development of posterior joint degeneration. Therefore, we believe that the results of this research, such as the stress on the intervertebral disc of the adjacent segment and the reaction force of the facet joint, can be used as a predictor of the development of potential lumbar degenerative diseases.

The ROM of a healthy adjacent segment increases to compensate for the decreased ROM of a degenerated segment (Park et al., 2015). Wang et al. (2018), introduced anterior vertebral osteophytes and decreased disc height, demonstrating that as the degeneration grade increased, the ROM of the adjacent segment also increased. Similarly, Park et al. (2015) conducted a study on cervical segments, considering both facet joint and disc degeneration, and found that as the degeneration grade increased, the ROM of the adjacent segment gradually increased. However, in our study, we observed almost no change in the ROM of the adjacent segment, which differs from the findings of previous studies. This discrepancy is attributed to the exclusion of disc degeneration and height reduction because our study focused solely on FJD. Therefore, we believe that disc degeneration may have a more significant impact on ROM changes in the adjacent segment compared to FJD.

In this study, we found no significant differences in spinal ROM, the maximum von Mises stress on the intervertebral disc, and the reaction force of the facet joint values between intact, L4/5 FJD, and L4/S1 FJD for the upper segments corresponding to L1-L3 FSU. As a result, we did not include the upper segments in the results of the present study.

As mentioned earlier, although little change was observed in the ROM of the adjacent segment in this study, we observed an increase in the load on both the disc and facet joint of the adjacent segment. Notably, the facet joint had a greater impact in both segments. This finding aligns with the effects of disc or FJD in the adjacent segments due to spinal degeneration. Consequently, in clinical practice, if disc and FJD occur simultaneously, the impact on the adjacent segment is expected to be greater, potentially accelerating additional degeneration. Previous studies have predominantly focused on lumbar intervertebral discs in the context of spinal degeneration, resulting in numerous research efforts related to disc degeneration (Pfirrmann et al., 2001; Schmidt et al., 2009; Fan et al., 2015; Ellingson et al., 2016). However, considering that spinal load is distributed between the lumbar intervertebral discs and facet joints is important. Therefore, research on facet joints is necessary to comprehensively understand spinal degeneration. This study adopted a novel approach as it exclusively examined lumbar FJD.

Several simplifications and assumptions were made in this study. Specifically, the investigation focused solely on degenerative changes in facet joints during spinal degeneration. Although various shape changes related to FJD, such as osteophytes and asymmetry, can occur, this study employed the number of onset segments and the gap and friction coefficients of the facet joint as parameters to explore changes in lumbar characteristics across the four grades of degeneration. The location of the onset segment with FJD was assumed to be limited to the most commonly affected L4–L5 FSU and L5–S1 FSU. Moreover, real patients with FJD often experience disc height reduction; however, for the purposes of this study, it was assumed that the disc height remained constant regardless of the facet joint gap. This decision was made to exclusively investigate the impact of FJD. Therefore, this study offers insights into the biomechanical characteristics of the lumbar region by considering FJD. However, further observational studies are necessary to validate the clinical changes caused by isolated lumbar FJD, as examined in this research.

There are also limitations in the modeling of the lumbar spine and the material properties applied. First of all, the author’s vertebrae are divided into cortical bone, cancellous bone, and posterior body, and each is assumed to have simple isotropic linear elastic material properties. However, when we look at the cervical vertebrae, we see that each part has different bone density and consequently different mechanical properties (Garay et al., 2022). And we see a non-uniform distribution of bone density throughout the vertebrae (Al-Barghouthi et al., 2020).

In addition, the cartilage and intervertebral discs of the facet joints are limited by the approach of implementing the facet joints under contact conditions among the various facet joint implementations in this study. However, the actual cartilage of the facet joints is a porous elastic model, and its mechanical properties change as the water content decreases and become stiffer during facet joint degeneration (Elmasry et al., 2017; Elmasry et al., 2018). The intervertebral disc model developed in this study is a hyperelastic material model, which has the limitation of simplicity. In recent years, various modeling methods have been used in intervertebral disc studies. One of them is an analysis using a porous elastic model (Volz et al., 2022). The porous elastic model models the biomechanical properties of intervertebral discs by considering the porosity and fluid flow in the disc. The model takes into account the interaction of liquids and solids inside the intervertebral disc and helps to understand the mechanisms of intervertebral disc degeneration. Cappetti et al. demonstrate the strong influence of geometric parameters in intervertebral disc modeling (Cappetti et al., 2016). Geometric parameters include the radius, thickness, status, curvature, position, and orientation of the intervertebral disc, which are said to affect the biomechanical properties of the intervertebral disc. To evaluate how each parameter affects the output, they used a sensitivity analysis method using Taguchi Orthogonal Array, which allows them to consider many parameters while minimizing the number of experiments, and quickly identify the parameters that have a significant impact on the output. This allows us to improve the accuracy of our modeling with less time and cost. In future research, we believe that by applying the advanced techniques in lumbar spine modeling and material properties mentioned above, we can simulate spinal behavior closer to the human body.

Another factor that was not monitored in this study but contributes significantly to pain is the disc bulging (Amirouche et al., 2015). Amirouche et al. describe segmental stiffness due to disc degeneration and the resulting degree of the disc bulging in cervical spine subjects. Further studies should consider not only facet joint degeneration but also intervertebral disc degeneration, so that the degree of the disc bulging can be utilized as a measure of spinal pain.

Among the various methods used to implement the FJs in this study, contact conditions were utilized (Mengoni, 2021). However, contact conditions cannot be applied when the contact surfaces are not in contact, such as during flexion (Cai et al., 2020). Additionally, this study did not consider changes in the properties of capsular ligament during FJD. Consequently, it does not have a significant impact on the ROM and intervertebral disc. Therefore, the FJD implementation method in this study was found to have no significant impact on the ROM and biomechanical characteristics of the intervertebral disc in flexion due to its simplification. In this study, four levels of degeneration were arbitrarily set by adjusting the gap and friction coefficient of the facet joint to 75, 50, 25, and 10% of the intact state (100%) for implementing FJD. However, considering that spinal load is distributed between the lumbar intervertebral discs and facet joints is important. Therefore, research on facet joints is necessary to comprehensively understand spinal degeneration. Recently, studies have been reported on the effects of facet joint parameters such as facet orientation (FO) and facet tropism (FT) on the lumbar spine (Teo et al., 2003; Kim et al., 2013; Li et al., 2020; Ke et al., 2021). The left and right facet joint angles of the vertebral body in the sagittal plane are referred to as FO, and the difference between the left and right angles is FT. The biomechanical effects of FT and FO on lumbar segmental stresses have been investigated, and it is reported that FT has the greatest effect on increasing intradiscal pressure and facet joint pressure (Kim et al., 2013; Ke et al., 2021). Studies investigating the association between FO and FT and recurrent lumbar disc herniation (rLDH) have found that FO and FT are associated with the development of rLDH, with decreased FO reported to be associated with an increased risk of rLDH and increased FT reported to be associated with an increased incidence of rLDH (Li et al., 2020). This suggests that facet joint parameters that were not considered in this study may also influence the lumbar spine. Therefore, this study is original in that it considered the parameter (gap size, friction coefficient) of lumbar FJD.

## 5 Conclusion

Overall, the results of this study showed that the decrease in the ROM of the onset segment due to FJD was caused by a decrease in the ROM of the entire segment and a decrease in the maximum von Mises stress of the intervertebral disc of the onset segment. However, the maximum von Mises stress on the IVD of the onset-adjacent segment during flexion and extension gradually increases as the level of degeneration becomes more severe. The facet joint reaction force decreased during the extension motion in the onset segment and then increased, whereas it increased during lateral bending and axial torsion. In contrast, the facet joint reaction force gradually increased in the adjacent segments.

The onset segment enters the stage of restabilization at Grade 4 degeneration and its instability is reduced further. However, the increased load on the intervertebral disc and facet joint in the adjacent segment may contribute to its instability. Therefore, inadequate neural control and compensatory lumbar muscles are thought to contribute to the degeneration of the adjacent segments in clinical scenarios.

## Data Availability

The original contributions presented in the study are included in the article/Supplementary material, further inquiries can be directed to the corresponding authors.
